# Functional Properties of *Dunaliella salina* and Its Positive Effect on Probiotics

**DOI:** 10.3390/md20120781

**Published:** 2022-12-15

**Authors:** Ivana Hyrslova, Gabriela Krausova, Iva Mrvikova, Barbora Stankova, Tomas Branyik, Hana Malinska, Martina Huttl, Antonin Kana, Ivo Doskocil

**Affiliations:** 1 Department of Microbiology and Technology, Dairy Research Institute Ltd., 160 00 Prague, Czech Republic; 2 Department of Microbiology, Nutrition and Dietetics, Czech University of Life Sciences Prague, 165 00 Prague, Czech Republic; 3 4th Department of Medicine-Department of Gastroenterology and Hepatology, First Faculty of Medicine, Charles University in Prague, 128 00 Prague, Czech Republic; 4 Department of Biotechnology, University of Chemistry and Technology, 162 00 Prague, Czech Republic; 5 Centre for Experimental Medicine, Institute for Clinical and Experimental Medicine, 140 21 Prague, Czech Republic; 6 Department of Analytical Chemistry, University of Chemistry and Technology, 162 00 Prague, Czech Republic

**Keywords:** adherence, algae, *Dunaliella salina*, cytokine, prebiotic

## Abstract

The unicellular green microalga *Dunaliella* is a potential source of a wide range of nutritionally important compounds applicable to the food industry. The aim of this study was to assess the effect of *Dunaliella salina* dried biomass on the growth and adherence of 10 strains of *Lactobacillus*, *Lacticaseibacillus,* and *Bifidobacterium*. The immunomodulatory, antioxidant, and cytotoxic effects of *D. salina* on human peripheral mononuclear cells and simulated intestinal epithelial cell lines Caco-2 and HT-29 were evaluated. Furthermore, the hypocholesterolemic effects of the microalgae on lipid metabolism in rats fed a high-fat diet were analyzed. The addition of *D. salina* biomass had a positive effect on the growth of nine out of 10 probiotics and promoted the adherence of three bifidobacteria strains to human cell lines. The antioxidant and immunomodulatory properties of *D. salina* were concentration-dependent. The inflammatory cytokines (TNF-α and IL-6) were significantly increased following *Dunaliella* stimulation at the lowest concentration (0.5% *w/v*). Eight week supplementation of *D. salina* to the diet of hypercholesteromic rats significantly decreased the serum concentrations of LDL-C, VLDL, IDL-B, and IDL-C. *D. salina* is not cytotoxic in intestinal cell models; it promotes adherence of selected bifidobacteria, it affords immunomodulatory and antioxidant effects, and its addition to diets may help decrease atherosclerosis risk factors.

## 1. Introduction

Microalgae have attracted more attention in recent years owing to their wide range of nutritionally important compounds for humans and animals, including polysaccharides, polyunsaturated fatty acids (PUFA), proteins, and antioxidants, such as carotenoids and phenolic compounds [1]. One of these microalgae is *Dunaliella* sp., which can accumulate large amounts of the β-carotene under appropriate conditions, in the range of 8–14% of its total dry weight [2]. β-Carotene produced by *D. salina* consists of both *cis* and *trans* isomers and a high antioxidant potential compared with synthetic β-carotene, which consists predominantly of *trans* isomers [3]. Xanthophylls (lutein and zeaxanthin) and polyphenols are other potential algal antioxidants. Studies on *Dunaliella* sp. have revealed that *Dunaliella* biomass or its extracts have a positive effect on the treatment of cardiovascular diseases and cancer, and they exhibit immunomodulatory and anti-inflammatory properties [3,4,5,6].

The whole range of compounds produced by *Dunaliella* sp., such as carotenoids (mainly β-carotene), glycerol, and cosmetic compounds, as well as its biomass, has industrial relevance as a source of valuable proteins [7]. Biomass, as well as carotenoids in lyophilized/dried form, is used in the food and feed industry as additives (coloring) or supplement for humans or animals [5,8]. *Dunaliella*, together with other microalgae, *Arthrospira* and *Chlorella*, have been approved by the Food and Drug Administration (FDA) as a food source with a Generally Recognized as Safe (GRAS) status [9]. However, regulations differ among countries. The GRAS status only applies to the USA. In the European Union (EU), the European Food Safety Authority (EFSA) decides on regulations pertaining to human food and animal feed. New foods, excluding genetically modified organisms (GMOs), are labeled as “novel food” and must undergo a safety assessment by the EFSA before being marketed [10]. In the case of microalgae in the EU, only *Arthrospira platensis*, *C. pyrenoidosa,* and *C. vulgaris* have been approved. Outside the USA, *Dunaliella salina* is considered safe in China and Canada [11,12].

Lactic acid bacteria (LAB) are widely used for the production of fermented foods or dietary supplements and are also part of the human and animal microbiota. Due to their long history of safe use in foods and their beneficial effects on human health, most LAB are used as probiotics [13]. Numerous studies have reported their health benefits, such as reducing hypertension or cholesterol, supporting the immune system, maintaining a healthy gut balance, and preventing diarrhea [14,15].

Polysaccharides, oligosaccharides, or polyphenols produced by microalgae are also studied for their possible prebiotic potential [16,17]. Prebiotics are described as substances whose selective fermentation affects the activity and/or composition of the GIT microbiota and, thus, has a positive effect on the host health [17,18]. The positive prebiotic effects of microalgae such as *C. vulgaris* and *Arthrospira* on the viability of LAB and bifidobacteria have been described by Hyrslova et al. [19,20], Ścieszka et al. [21], and Beheshtipour et al. [22]. However, information regarding the growth-promoting effect of *D. salina* biomass on probiotic strains and their adherence to human cells is lacking. Therefore, the aim of the present study was to evaluate the functional properties of *D. salina* dried biomass and its possible synergistic effects on probiotics (bifidobacteria and LAB). This will contribute to the development of new functional foods or food supplements for humans or animals. First, the influence of *D. salina* on the growth and adherence of selected probiotics was determined. Furthermore, cytotoxic, immunomodulatory, and antioxidant effects were assessed. *Dunaliella* is a rich source of carotenoids and other bioactive compounds that can help in the prevention or treatment of obesity and associated diseases [1,23]. Second, the hypocholesterolemic effect of *D. salina* biomass in a Prague hereditary hypercholesterolemic rats (PHHC) model with diet-induced hypercholesterolemia was evaluated [24].

## 2. Results

### 2.1. Bacterial Growth in the Presence of D. salina Biomass

The effect of *D. salina* biomass on the growth of *Lactobacillus, Lacticaseibacillus*, and *Bifidobacterium* strains was evaluated on the basis of the bacterial counts and pH value after 24 h of fermentation (Table 1). The cell counts of most strains ranged between 8 and 9 log CFU/mL after 24 h of fermentation, with the exception of *L. rhamnosus* CCDM 466. In this strain, the cell count viability increased slightly to 6 log CFU/mL after 24 h of incubation.

### 2.2. Cytotoxic Assay

The cytotoxic effect of *D. salina* biomass on human adenocarcinoma cell lines Caco-2 and HT-29 was evaluated using an MTT (3-(4,5-dimethylthiazol-2-yl)-2,5 diphenyltetrazolium bromide) assay. The cytotoxic responses of the tested cell lines after treatment with different content of *D. salina* biomass were different according to the type of cell lines used. The IC_50_ value of the goblet cells of the HT29 cell line was higher than 512 μg/mL, which indicates no toxicity. Instead, the Caco-2 cells appear to be more sensitive to *D. salina* biomass, where the IC_50_ value reached 192 μg/mL.

### 2.3. Adherence

Caco-2 and HT-29 cell lines were used as models of the intestinal epithelium for the in vitro determination of the influence of *D. salina* biomass on the adherence ability of the *Lactobacillus*, *Lacticaseibacillus*, and *Bifidobacterium* strains. Both contents of *D. salina* (64 and 125 μg/mL) had a positive effect on the adherence of *B. animalis* subsp. *lactis* BB12 to the HT-29 cell line and *Bifidobacterium breve* CDDM 562 to the Caco-2 cell line (Table 2). The highest content of *D. salina* biomass supported the adherence ability of *B. animalis* subsp. *lactis* CCDM 93 in both the tested cell lines. The opposite effect was observed for the remaining strains. The addition of *D. salina* biomass (125 μg/mL) decreased adherence to *L. acidophilus* CCDM 151 approximately 65%.

### 2.4. Immunomodulatory Effect

The immunomodulatory responses of hPBMCs after treatment with three different content of *D. salina* biomass were compared on the basis of the production of proinflammatory and regulatory cytokines (IL-4, IL-10, IL-17, IL-6, and TNF-α). Cytokine levels were determined by multiplex analysis. The production of TNF-α and IL-6 was significantly higher after stimulation with the lowest content of *D. salina* (0.5 % *w/v*). The levels of these cytokines decreased with increasing content of *D. salina* (Table 3). The production of the remaining cytokines (IL-6, IL-10, and IL-17) by mononuclear cells following *D. salina* biomass stimulation was not significantly altered.

### 2.5. Antioxidant Assay

**Figure 1:**
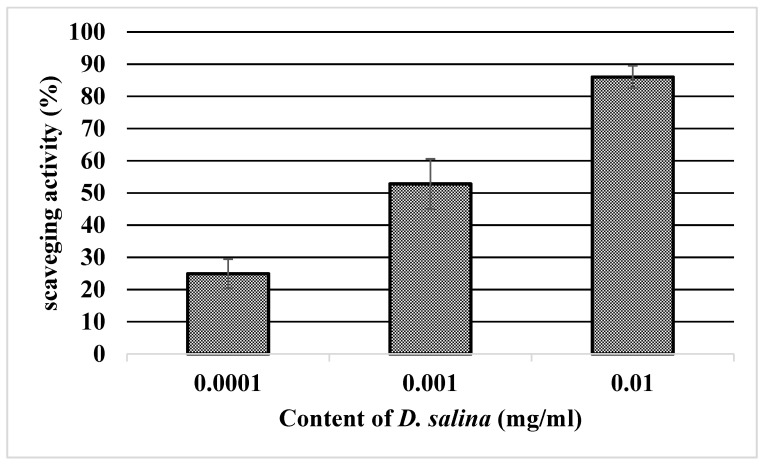
Free-radical-scavenging (DPPH) activity of *D. salina* biomass.

The antioxidant activity of *D. salina* biomass was tested using the DPPH assay. The free-radical-scavenging activity showed an increasing antioxidant effect with increasing content of microalgae (Figure 1).

### 2.6. Hypocholesterolemic Effect

**Figure 2:**
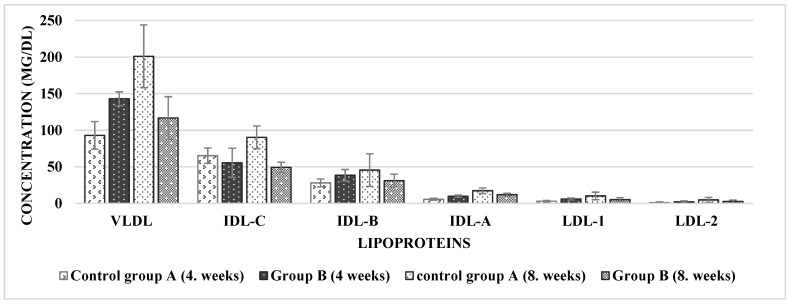
Serum lipid profiles in a rat model after feeding on a high-cholesterol diet supplemented with *D. salina.* VLDL, very-low-density lipoprotein; LDL, low-density lipoprotein; IDL, intermediate-density lipoprotein; Group A, fed the hypercholesterolemic diet; Group B, fed the hypercholesterolemic diet + *D. salina* biomass. Values are presented as the mean ± standard deviation, n = 6.

In the present study, the influence of *D. salina* dried biomass on lipid metabolism in male rats with hypercholesterolemia was tested. The concentrations of lipid parameters, such as TAG, total cholesterol (TC), lipoproteins (LDL, IDL, HDL, and VLDL), and aminotransferases (ALT and AST) were determined in sera and tissues at the midpoint and at the end of the experiment (eight weeks). In the group fed on the diet with *D. salina*, the levels of TC and LDL-C significantly decreased (*p* < 0.05) after 8 weeks of treatment compared with that of the control group A (Table 4). The same decreasing effect was observed for VLDL, IDL-C, and IDL-B (*p* < 0.05) (Figure 2), as well as for levels of TAG (aorta and serum), HDL-C, and aminotransferases.

## 3. Discussion

Algal species, such as *Arthrospira* sp., *Chlorella* sp., and *D. salina*, have been used in the food and pharmacological industries because they are rich in proteins, polysaccharides, and other bioactive compounds [1,25]. The chemical profile of these compounds may differ depending on the algal species, cultivation conditions, growth stage, and other factors. *Dunaliella* is more easily digested by humans and animals than other microalgae due to their lack of rigid cellulose cell walls [2,9]. Additionally, 50–80% of proteins in the dried biomass of *Dunaliella* comprise quality essential amino acids (EAAs) for human requirements [26]. This EAA composition is comparable to that of soybeans or chlorella [27]. Microalgal biomass is a promising alternative source of prebiotics because of its high oligosaccharide, polysaccharide, and phenolic compound content [28,29]. Nevertheless, substances or food ingredients for acceptance as prebiotics must fulfil several criteria described by Gibson et al. [30]. They must be at least partially resistant to GIT conditions (enzymatic activity and low pH) and utilized in the intestine. Furthermore, they also should be fermented by intestinal microbiota and selectively stimulate their growth and/or activity, thus influencing the health and wellbeing of the host [17].

The influence of *Dunaliella* extract/biomass upon the viability of lactic acid bacteria and bifidobacteria was described for the first time in this work. Nevertheless, the positive effect of Chlorella and Arthrospira on the growth of these bacteria was proven in a range of studies [20,21,22,23]. *Arthrospira platensis* promoted the growth of *Lacticaseibacilus casei*, *Streptococcus thermophilus*, *Lactobacillus acidophilus*, or bifidobacteria. In our previous studies, we proved the positive effect of *Chlorella vulgaris* biomass on viability of *B. animalis* subsp. lactis BB-12 and CCDM 93 [20,21]. Zhou et al. [31] tested the aqueous extracts of *Arthrospira*, *Chlorella*, and *P. tricornutum* with a high content of carbohydrates, proteins, and phenolic compounds obtained using pressurized liquid extraction (PLE). PLE extract promoted the growth of probiotic *Lacticaseibacillus casei* BL23 and *B. animalis* subsp. lactis BB12 and inhibited some of the foodborne pathogens, such as *Listeria innocua* CECT 910, *Salmonella enterica* CECT 4138, *Staphylococcus aureus* CECT 86, and *Escherichia coli* CECT 99. In the present study, the growth-promoting effect of *D. salina* on 10 different strains of *Lactobacillus*, *Lacticaseibacillus*, and *Bifidobacterium* was investigated. The growth-promoting effect of *D. salina* biomass was observed in all strains. The cell count increased by approximately 4–5 log CFU/mL after 24 h of fermentation. One exception was *L. rhamnosus* CCDM 466, which exhibited weaker growth. It is likely that changes in growth could be caused by different metabolic requirements, energy sources, or antimicrobial activities of compounds from *D. salina*. The antimicrobial properties of extracts or particular compounds obtained from *D. salina* have been evaluated in multiple studies [32,33,34]. Herrero et al. [34] proved the antimicrobial activity of *D. salina* extract gained using different solvents (hexane, petroleum ether, hexane, and water) against undesirable food industry microorganisms, such as *Escherichia coli*, *Staphylococcus aureus*, and *Candida albicans*. They also identified 15 volatile compounds and fatty acids that could have been responsible for the antimicrobial activity [7,34].

Adherence of probiotic bacteria to the intestinal mucosa is an important prerequisite for the colonization of the intestinal epithelium and inhibition of pathogenic microorganisms [35]. Therefore, we tested the influence of *D. salina* biomass on the adhesion abilities of selected lactic acid strains and bifidobacteria. Our results showed that the influence of *D. salina* biomass on adherence is strain-specific and depends on the concentration of microalgae. Three strains from the genus *Bifidobacterium* (*B. breve* 562, *B. animalis* subsp. *lactis* BB-12, and CCDM 93) exhibited improve adhesion to HT-29 and Caco-2 after the addition of *D. salina*. Therefore, *D. salina* had a positive effect on the adherence ability of these strains. In contrast, adherence to the lactobacilli decreased. Prebiotics have been shown to have either negative or positive effects on the adherence of specific bacterial strains [36,37]. Therefore, it is important to identify the correct prebiotic for specific probiotic strains to achieve the best synbiotic effect [35]. Jafari et al. [38] tested the anti-adherent and antimicrobial effects of *D. salina* and *Chlorella vulgaris* extract against *Streptococcus mutans* PTCC 1683 causing dental caries. The extract of *D. salina* (2 mg/m) significantly inhibited the production of biofilm formed by *S.mutans*, and the antimicrobial activity against this strain was observed. The antibiofilm activity of *D. salina* extract might be connected to inhibition of water-insoluble glucans induced by activity of glucosyltransferases (GTF) [38].

The immune system is divided into innate and adaptive systems. The innate immune system is an immediate nonspecific response mediated by macrophages, natural killer (NK) cells, and dendritic cells [39]. Adaptive immunity is an antigen-specific defense system connected by the activity of B and T cells. Th1 cells are important for cell-mediated responses related to cytotoxic T cells and macrophages. Th1 cells secrete TNF-α, IL-12, IL-2, or IFN-γ to induce cellular immunity and are involved in defense processes against intracellular microbes [40]. Th2 cells influence the protection against parasites and produce IL-4, IL-5, and IL-10 to induce immune responses. Th17 cells are involved in the host defense against bacteria and fungi. Th17 cells secrete IL-6, IL-17, and IL-22 [39,40]. Our results showed that three concentrations of aqueous *D. salina* solution (0.5%, 1.0%, and 3.0% *w/v*) influenced the production of IL-6 and TNF-α. The highest levels of these cytokines were observed after stimulation with the lowest content of *D. salina*. Increased TNF-α levels induce higher production of IL-6 to maintain the Th1/Th2 balance [41]. Goyal et al. [42] tested the immunomodulatory activity of the ethyl acetate fraction of crude EPS produced by *D. salina* against peripheral blood mononuclear cells (PBMCs) and RAW 264.7 macrophages. In contrast to our results, the level of TNF-α secreted by PBMCs increased after treatment with higher concentrations of *D. salina* extract (750 and 1000 µg/mL) [43]. The difference in TNF-α secretion may have been caused by the different determination methods used. Cytokine levels are usually evaluated using flow cytometry or ELISPOT [40,41,42,43]. In our study, Luminex multiplex assays were used for simultaneous quantitative determination of multiple human cytokine concentrations in cell culture supernatants, sera, and plasma. Another reason for the differences observed between these studies may be the different sample types used; in the current study, we tested an aqueous solution of biomass, whereas Goyal et al. [42] used the ethyl acetate fraction of *D. salina*. Nevertheless, both studies showed that the immunomodulatory activity of the extract or biomass of *D. salina* is dose-dependent.

The high antioxidant content in *D. salina* means that his microalgal species may be able to scavenge reactive oxygen species (ROS) [44,45]. ROS are associated with the aging process and the pathogenesis of cardiovascular diseases, cancer, and diabetes [4,46]. The free-radical scavenging activity of *D. salina* biomass showed an increased antioxidant effect depending on the concentration of microalgae. Singh et al. [4] described the change in antioxidant and cytotoxic activities of carotenoids produced by *D. salina* as a result of increasing salinity, nitrogen, and temperature stress. The results showed that carotene content and scavenging activity were positively correlated under various stress conditions. Cytotoxicity in MCF-7 cells also increased with carotenoids accumulation [4]. In the present study, the cytotoxic response induced by *D. salina* biomass on the human adenocarcinoma cell lines Caco-2 and HT29 was different. Caco-2 cells were twice as sensitive (IC_50_ = 191.8 μg/mL) as HT-29 cells (IC_50_ > 512 μg/mL). Extracts with IC_50_ values >90 µg/mL are considered nontoxic [47]. The IC_50_ limit for both cell lines was >90 µg/mL. Differences in IC_50_ values between tested cell lines after treatment by *D. salina* biomass could have been caused by different phenotypes of the cells present in intestinal epithelium. Caco-2 cells are enterocytes and HT-29 cells are goblet cells producing mucin [48]. Senousy et al. [49] studied anticancer potential against four different cancerous cell lines (Caco-2, MCF-7, HEpG-2, and PC-3) and cytotoxic effect against human PBMCs from healthy donors of cyanobacteria and two strains of microalgae (*Dunaliella* sp. HSSASE13 and *Chlorella sorokiniana* HSSASE17). The IC_50_ of tested *Dunaliella* sp. HSSASE13 was 266 ± 0.001 (µg/mL) on hPBMCs. Nevertheless, the values of IC_50_ for cancer cell lines were significantly lower. In the case of Caco-2 cancer cells (also used in this study), the IC_50_ was 106.8 ± 0.3 µl/mL. Zamani et al. [44] also demonstrated that *D. salina* extract exhibit strong antioxidant and anticancer effects on HeLa and MC7 human cell lines in a time- and dose-dependent manner.

*D. salina* and its extracts show promise for the prevention and treatment of obesity and related diseases [50]. *Dunaliella*, mainly because of its high β-carotene content, can protect LDL against oxidation, thereby preventing atherosclerosis. Shaish et al. [51] observed a positive effect of *Dunaliella bardawil* administration on the inhibition of LDL oxidation and reduction of plasma TG, cholesterol, and HDL levels. Phytosterol compounds contained in the microalgae biomass influence lipid metabolism owing to their cholesterol-lowering activity [51]. Phytosterols reduce LDL absorption through the displacement of cholesterol from lipid micelles during the intestinal absorption of phytosterols, but do not affect the levels of HDL and total cholesterol [16,40]. Therefore, in the present study, we aimed to test the influence of *D. salina* biomass on lipid metabolism in hypercholesterolemic male rats. Additionally, the concentrations of atherosclerosis risk factors, such as TAG, total cholesterol (TC), lipoproteins (LDL, IDL, HDL, and VLDL), and aminotransferases (ALT and AST), were determined in sera and tissues at the midpoint and at the end of the experiment (8 weeks). The addition of *D. salina* biomass significantly decreased the concentrations of LDL-C, VLDL, IDL-B, and IDL-C in the sera of treated hypocholesterolemic rats after 8 weeks. The addition of *D. salina* along with a high-cholesterol diet led to significantly decreased total serum cholesterol and triglyceride levels after 2 months in comparison with that of rats fed only a high-cholesterol diet. Bansal and Jaswal [3] obtained similar results. No change in liver function enzyme activities (ALT and AST) were observed after administration of *D. salina*, in accordance with the study by El-Baz et al. [52], who also examined the safety of *D. salina* as a supplement using in vivo models (mice, rats).

## 4. Materials and Methods

### 4.1. Microorganisms

Eight bacterial strains from the genera *Lactobacillus*, *Lacticaseibacillus*, and *Bifidobacterium* were selected from the Culture Collection of Dairy Microorganisms Laktoflora^®^ (Tabor, Czech Republic): CCDM 146, 466 *Lacticaseibacillus rhamnosus*, CCDM 364 *Lactobacillus bulgaricus*, CCDM 151 *Lactobacillus acidophilus*, CCDM 562 *B. breve*, CCDM 232 *B. longum* subsp. *infantis*, CCDM 219 *B. longum* subsp. *longum*, and CCDM 93 *B. animalis* subsp. *lactis*. Two commercial strains, *Bifidobacterium animalis* subsp. *lactis* BB-12^®^ (Chr. Hansen, Hørsholm, Denmark), and *Lactobacillus casei* Lafti L-26 (DSM Food Specialties, Leeuwarden, the Netherlands), were used as the controls. Prior to each analysis, bacterial cells were transferred twice to fresh De Man–Rogosa–Sharpe (MRS, pH 6.2/5.7) broth (Merck, Darmstadt, Germany), with L-cysteine hydrochloride (Merck) for bifidobacteria, and cultivated under anaerobic conditions at 37 °C for 18 h. *D. salina* dried biomass was obtained from a commercial network (Plankton Australia Pty Ltd., Alexandria, Australia).

### 4.2. Bacterial Growth in the Presence of Dunaliella salina

The bacterial growth was assessed according to the method described by Hyrslova et al. [19]. The basal medium was supplemented with 2 g/L *Dunaliella* dried biomass (pH 6.5). Strains were grown overnight, separated from the medium by centrifugation (6000× *g*, 7 min), washed with sterile saline solution, and resuspended to a final concentration of approximately 10^4^ colony forming units (CFU)·mL^−1^. All media were inoculated with 1.0% (*v/v*) bacterial suspension and cultivated in anaerobic jars (Merck) at 37 °C for 24 h. The results were obtained from three independent measurements.

### 4.3. DPPH Free-Radical-Scavenging Activity

The DPPH radical-scavenging assay was performed according to the method described by Krausova et al. [53]. The reaction mixtures were formulated by adding 1 mL of 0.2 mmol/L DPPH in methanol to 1 mL of *Dunaliella* biomass suspension (0.1 g/10 mL, 0.01 g/10 mL, and 0.001 g/10 mL). The mixtures were incubated for 30 min at 25 °C in the dark, and the color change was measured at 517 nm using an ONDA V-10 Plus spectrophotometer (Giorgio Bormac, Carpi, Italy). The DPPH free radical-scavenging activity (%) of the tested samples was determined using Equation (1)

DPPH free-radical-scavenging activity (%) = (1 − (As − Ac)/Ab) × 100,(1)

where As is the absorbance of the sample with DPPH, Ac is the absorbance of the control sample (sample with methanol), and Ab is the absorbance of distilled water with DPPH.

### 4.4. Adherence

The influence of *D. salina* biomass on the adherence ability of selected probiotic strains to human colon adenocarcinoma cell lines (Caco-2 and HT-29) was determined using a method previously described by Musilova et al. [54] with minor modifications. The cell lines were cultured in Dulbecco’s modified Eagle’s medium (DMEM) supplemented with 10% fetal bovine serum, 1% nonessential amino acids, 100 μg/mL penicillin, and 100 μg/mL streptomycin (Sigma-Aldrich, Steinheim, Germany) at 37 °C in a humidified atmosphere containing 5% CO_2_. Caco-2 and HT29 cells were seeded in 24-well culture plates at a density of 10^4^ cells/mL. The tested strains (approximately 10^7^ CFU/ml) with *D. salina* suspensions of 64 and 125 μg/mL were added to the wells and incubated at 37 °C for 90 min. The cell layers were then gently washed thrice with sterile PBS to remove nonadherent bacteria. Finally, 300 μL of 1 % Triton-X100 was added to each well for 30 s, followed by the addition of 700 μL of PBS. After incubation, the cells were detached from the wells. The remaining suspensions with viable adhered strains were diluted and cultivated on appropriate agar plates (Merck, Germany) at 37 °C for 3 days under anaerobic conditions. The adhesion ability of the selected strains toward HT-29/Caco-2 cells was expressed as the percentage of adhered strains compared to the total count.

### 4.5. Animals and In Vivo Study Design

The hypocholesterolemic effect of *D. salina* biomass was evaluated using a male Prague hereditary hypercholesterolemic rat (PHHC) model with diet-induced hypercholesterolemia (PHHC; Albert Weber-SEMED, Praha, Czech Republic), according to the previous study of Hyrslova et al. [20], with some modifications. After acclimatization to laboratory conditions for 2 weeks, 8 week old rats were randomly divided into two groups (A and B) (n = 10). A hypercholesterolemic diet (Albert Weber-SEMED, Prague, Czech Republic) fortified with 2.0% (*v/w*) cholesterol was served ad libitum. Group A (control) was only fed the hypercholesterolemic diet. Group B was fed the hypercholesterolemic diet and received 600 µL of 0.5% (*w/v*) Dunaliella biomass suspension by oral gavage, daily for 4 and 8 weeks. Following anesthesia (zoletil 5 mg/kg body weight, Virbac, Carros, France), a decapitation was used for euthanasia of animals in the postprandial state. Tissue samples and serum aliquots were stored at −80 °C for further analysis. All presented experiments with laboratory animals were in conformity with the Animal Protection Law of the Czech Republic (311/1997 Coll.) in compliance with the European Community Council recommendations (86/609/ECC) for the use of laboratory animals. The in vivo study was also approved by the ethical committee of the Ministry of Education, Youth, and Sports (jMSMT-46654/2015-8).

### 4.6. In Vitro Cytotoxicity Assay

The cytotoxic effect of D. *salina* biomass was determined according to the method described by Hyrslova et al. [55], with some modifications. Caco-2 (ATCC HTB-37) and HT29 (ATCC HTB 38) cell lines derived from human adenocarcinoma (American Type Culture Collection, Rockville, MD, USA) were cultivated in Eagle’s minimal essential medium (EMEM, Sigma-Aldrich, Steinheim, Germany) supplemented with essential substances and antibiotics at 37 °C with 5% CO_2_. The viability of cells was evaluated using the MTT (3-(4,5-dimethylthiazol-2-yl)-2,5 diphenyltetrazolium bromide, Sigma-Aldrich) test. Human cell lines were seeded in 96-well plates at a density of 2.5 × 10^3^ cells/mL. After 24 h, the samples of biomass *D. salina* were added at different concentrations from 16 to 512 μg/mL and cultivated for 72 h. Subsequently, the medium containing *D. salina* biomass was replaced by EMEM supplemented with the MTT reagent (1 mg/mL) and incubated for 2 h. Subsequently, the medium was removed, and the final intracellular formazan product was dissolved with 100 μL of dimethyl sulfoxide. The absorbance was measured at 555 nm using a Tecan Infinite M200 spectrometer (Tecan Group, Männedorf, Switzerland). The cytotoxic effect (IC_50_ %) of *D. salina* biomass on Caco-2 and HT29 cell lines was assessed/ evaluated using Magellan™ software (Tecan Group, Grödig, Austria). The results are values from three different measurements.

### 4.7. Biochemical Parameters

Plasma concentrations of total cholesterol and triglycerides (TAG) were determined using enzymatic colorimetric methods (Boehringer, Mannheim, Germany). High-density lipoprotein cholesterol (HDL-C) was measured in the supernatant following the precipitation of lipoprotein B using phosphotungstic acid/Mg^2+^ (Merck, Darmstadt, Germany). Low-density lipoprotein cholesterol (LDL-C) levels were calculated according to the Friedewald formula. To determine TAG and cholesterol levels in the aorta, samples were extracted in a chloroform/methanol mixture. The resulting pellet was dissolved in isopropyl alcohol, and the TAG content was determined using an enzymatic assay (Erba-Lachema, Brno, Czech Republic). The catalytic activities of alanine aminotransferase (ALT) and aspartate aminotransferase (AST) in the sera were measured using commercial analytical methods according to the International Federation of Clinical Chemistry and Laboratory Medicine using an analyzer (Unicel DxC 880i; Beckman Coulter, Brea, CA, USA).

### 4.8. Immunomodulatory Assay

The immunomodulatory effect of *D. salina* biomass was assessed according to the method described by Hyrslova et al. [19], with some modifications. Eight samples of blood from healthy adults were ordered from the Blood Transfusion Center of General Faculty Hospital (Prague, Czech Republic) for the isolation of human peripheral blood mononuclear cells (hPBMCs) via Ficoll–Hypaque gradient separation. Following separation and purification, the final concentration of hPBMCs was adjusted to 10^7^ cells/mL. Mononuclear cells (0.1 mL) were stimulated in an X-vivo medium (Cambrex, East Rutherford, NJ, USA) with 0.1 mL of 0.5%, 1.0%, or 3.0% aqueous solution of *D. salina* biomass at 37 °C for 3 days. The total volume of the solution was 1 mL. The concentrations of selected cytokines after hPBMC stimulation with aqueous solutions of *D. salina* biomass were evaluated using a Fluorokine MAP Human Base Kit A (R&D Systems, Minneapolis, MN, USA) for interferon (IFN)-γ, interleukin (IL)-4, IL-6, IL-10, IL-17, and tumor necrosis factor (TNF)-α, by multiplex analysis using a Luminex 200 Analyzer (Luminex Corp., Austin, TX, USA). The concentration of cytokines produced by hPBMCs was assessed using Luminex IS 2.3 (Luminex Corp., Austin, TX, USA). The results were obtained from three independent measurements.

### 4.9. Statistical Analysis

The analysis of variance (ANOVA) was used when assumptions (homoscedasticity and normality of data distribution) were met. These assumptions were tested using the Levene and Shapiro–Wilk tests. In the case of heteroscedasticity, the Mann–Whitney test was used for comparing two individual groups, and the Kruskal–Wallis test followed by Dunn’s test was used as the nonparametric alternative of ANOVA. Statistica 13.1 (StatSoft, Inc., Tulsa, OK, USA) and Real Statistics Resource Pack (Release 7.2, Charles Zaiontz, www.real-statistics.com) were used to perform the tests. The immunomodulatory effect was evaluated using ANOVA with a post hoc least significance difference test (LSD) for multiple comparisons. All tests were performed with statistical significance set at *p* < 0.05. Statistical analysis of the cytotoxic effect was performed using Magellan^TM^ (Tecan Group, Grödig, Austria) and Microsoft Office Excel 2013 (Microsoft, Redmond, WA, USA), using data from three different experiments.

## 5. Conclusions

In summary, although *D. salina* is not as popular as *Chlorella* and *Arthrospira,* it has many beneficial properties for the development of new functional foods with probiotics and novel therapeutics. *D. salina* biomass demonstrated a positive growth effect on probiotics and promoted the adherence of *B. breve* 562, B. *animalis* subsp. l*actis* BB-12, and CCDM 93 on human cell lines. Most of the functional properties of *D. salina* biomass tested in the present study, such as the antioxidant, cytotoxic, and immunomodulatory effects, were dose-dependent. In addition to the concentration, these properties are influenced by the content of bioactive compounds and chemical composition of *D. salina* biomass, which depends on the cultivation conditions (saline, temperature, and nitrogen). Therefore, details of the chemical composition of *Dunaliella* used in the experiments are among the limitations of this study. The addition of *D. salina* to the diet of hypercholesterolemic rats significantly decreased the concentrations of LDL-C, VLDL, IDL-B, and IDL-C in sera after 8 weeks, thereby helping to decrease atherosclerosis risk factors.

## Figures and Tables

**Table 1:** Bacterial cell counts and pH after 24 h cultivation.

Bacterial Strains	Cell Counts (log CFU/mL)	pH
BB12	8.78 ± 0.07 ^c^	5.21 ± 0.01 ^a^
Lafti L-26	8.70 ± 0.09 ^c^	5.31 ± 0.00 ^b^
364	9.00 ± 0.04 ^d^	5.31 ± 0.02 ^b^
466	5.88 ± 0.15 ^a^	5.36 ± 0.01 ^c^
562	9.00 ± 0.31 ^c^	5.27 ± 0.04 ^b^
151	8.60 ± 0.11 ^c^	5.27 ± 0.01 ^b^
232	8.30 ± 0.24 ^b^	5.19 ± 0.01 ^a^
219	8.74 ± 0.04 ^c^	5.33 ± 0.05 ^b^
93	8.78 ± 0.07 ^c^	5.21 ± 0.03 ^a^
146	8.65 ± 0.17 ^c^	5.33 ± 0.01 ^b^

Values are the means of triplicate measurements ± standard deviation. ^a,b,c,d^ Data with different superscript letters differ significantly (*p* < 0.05).

**Table 2:** Difference of adherence (%) of *Lactobacillus*, *Lacticaseibacillus*, and *Bifidobacterium* strains in the presence of *D. salina* biomass compared to control.

	Content of *D. salina*			
	125 μg/mL		64 μg/mL	
Bacterial strains	Caco-2	HT-29	Caco -2	HT-29
219	−21.1 ± 10.9	−26.1.0 ± 7.0	−40.3 ± 9.2	−15.0 ± 5.5
BB12	−30.7 ± 11.4	+26.5 ± 15.5	−25.7 ± 13.5	+23.7 ± 16.9
93	+1.4 ± 10.3	+6.0 ± 3.7	− 9.7 ± 3.9	−14.6 ± 9.7
146	−15.3 ± 12.1	−26.1 ± 4.6	−7.1 ± 12.6	−28.2 ± 12.5
232	−19.3 ± 6.2	−3.0 ± 6.5	−10.6 ± 9.4	−22.0 ± 7.8
364	−14.9 ± 5.4	+2.5 ± 9.3	−8.6 ± 6.0	−22.2 ± 12.3
Lafti L−26	−34.6 ± 17.2	−36.2 ± 8.4	−2.1 ± 7.0	−53.3 ± 11.3
562	+4.5 ± 9.2	−21.2 ± 8.4	+7.0 ± 15.0	−33.7 ± 6.4
151	−65.0 ± 11.2	−66.8 ± 1.3	−14.1 ± 14.2	−35.8 ± 2.8
466	−22.2 ± 2.9	−10.1 ± 7.9	−19.8 ± 12.9	−17.2 ± 8.9

Values are the mean ± standard deviation. − decreased adherence; + increased adherence.

**Table 3:** Cytokine production by mononuclear cells following *D. salina* stimulation.

Concentration of *D. salina* (*w/v*)	Cytokines (pg/mL)				
	TNF-α	IL-4	IL-6	IL-10	IL-17
0.5%	51.2 ± 13.5 ^d^	4.8 ± 1.3 ^b^	329.6 ± 146.4 ^d^	4.1 ± 1.8 ^b^	1.8 ± 0.7 ^b^
1.0%	23.02 ± 6.7 ^c^	6.26 ± 2.5 ^b^	179.70 ± 114.7 ^c^	3.5 ± 1.5 ^b^	1.4 ± 0.8 ^b^
3.0%	8.3 ± 6.43 ^b^	7.32 ± 2.6 ^b,c^	13.65 ± 5.2 ^b^	3.6 ± 1.1 ^b^	4.1 ± 1.8 ^c^
Control	4.1 ± 0.8 ^a^	0.4 ± 0.1 ^a^	5.1 ± 0.7 ^a^	0.1 ± 0.0 ^a^	0.1 ± 0.0 ^a^

TNF, tumor necrosis factor; IL, interleukin. Values are presented as the mean ± standard deviation, n = 8. ^a,b,c,d^ Data with different superscript letters differ significantly (*p* < 0.05).

**Table 4:** TAG accumulation in tissues, serum lipid concentrations, and enzyme aminotransferases.

Tested Group	Periods	Biochemical Parameters						
		TAG Aorta (μmol/g)	TC (mmol/L)	HDL-C (mmol/L)	LDL-C (mmol/L)	TAG (mmol/L)	ALT (ukat/L)	AST (ukat/L)
Group A (control)	4 weeks	0.47 ± 0.06 ^A^	8.62 ± 0.58 ^A^	0.70 ± 0.08 ^A^	6.50 ± 0.69 ^A^	3.16 ± 0.79 ^A^	1.67 ± 0.12 ^A^	3.83 ± 0.15 ^A^
Group B		0.95 ± 0.49 ^A^	9.32 ± 0.42 ^A^	0.72 ± 0.07 ^A^	7.71 ± 0.58 ^B^	2.10 ± 0.38 ^B^	1.78 ± 0.15 ^A^	3.94 ± 0.66 ^A^
Group A (control)	8 weeks	1.44 ± 0.15 ^A^	12.28 ± 1.46 ^A^	0.71 ± 0.04 ^A^	9.9 ± 1.84 ^A^	2.40 ± 0.45 ^A^	2.15 ± 0.40 ^A^	4.41 ± 0.45 ^A^
Group B		1.47 ± 0.09 ^A^	8.77 ± 1.28 ^B^	0.63 ± 0.08 ^A^	7.1 ± 1.20 ^B^	2.02 ± 0.48 ^A^	1.75 ± 0.18 ^A^	3.71 ± 0.61 ^A^

TC, total cholesterol; ALT, alanine aminotransferase; AST, aspartate aminotransferase. Group A, fed the hypercholesterolemic diet; Group B, fed the hypercholesterolemic diet + *D. salina* biomass. Values represent the mean ± standard deviation, n = 6. Data with different superscript letters (^A, B^) are significantly different (*p* < 0.05).

## Data Availability

All data are presented in the paper.
